# Empowering the people: Development of an HIV peer education model for low literacy rural communities in India

**DOI:** 10.1186/1478-4491-6-6

**Published:** 2008-04-18

**Authors:** Koen KA Van Rompay, Purnima Madhivanan, Mirriam Rafiq, Karl Krupp, Venkatesan Chakrapani, Durai Selvam

**Affiliations:** 1 Sahaya International Inc., Davis, USA; 2 University of California, Davis, USA; 3 University of California, School of Public Health, Berkeley, USA; 4 Indian Network for People living with HIV/AIDS, Chennai, India; 5 Rural Education and Action Development (READ), Vilandai, Andimadam Post, Tamil Nadu, India

## Abstract

**Background:**

Despite ample evidence that HIV has entered the general population, most HIV awareness programs in India continue to neglect rural areas. Low HIV awareness and high stigma, fueled by low literacy, seasonal migration, gender inequity, spatial dispersion, and cultural taboos pose extra challenges to implement much-needed HIV education programs in rural areas. This paper describes a peer education model developed to educate and empower low-literacy communities in the rural district of Perambalur (Tamil Nadu, India).

**Methods:**

From January to December 2005, six non-governmental organizations (NGO's) with good community rapport collaborated to build and pilot-test an HIV peer education model for rural communities. The program used participatory methods to train 20 NGO field staff (Outreach Workers), 102 women's self-help group (SHG) leaders, and 52 barbers to become peer educators. Cartoon-based educational materials were developed for low-literacy populations to convey simple, comprehensive messages on HIV transmission, prevention, support and care. In addition, street theatre cultural programs highlighted issues related to HIV and stigma in the community.

**Results:**

The program is estimated to have reached over 30 000 villagers in the district through 2051 interactive HIV awareness programs and one-on-one communication. Outreach workers (OWs) and peer educators distributed approximately 62 000 educational materials and 69 000 condoms, and also referred approximately 2844 people for services including voluntary counselling and testing (VCT), care and support for HIV, and diagnosis and treatment of sexually-transmitted infections (STI). At least 118 individuals were newly diagnosed as persons living with HIV (PLHIV); 129 PLHIV were referred to the Government Hospital for Thoracic Medicine (in Tambaram) for extra medical support. Focus group discussions indicate that the program was well received in the communities, led to improved health awareness, and also provided the peer educators with increased social status.

**Conclusion:**

Using established networks (such as community-based organizations already working on empowerment of women) and training women's SHG leaders and barbers as peer educators is an effective and culturally appropriate way to disseminate comprehensive information on HIV/AIDS to low-literacy communities. Similar models for reaching and empowering vulnerable populations should be expanded to other rural areas.

## Background

Despite increased efforts in recent years and widely varying prevalence estimates, the HIV epidemic in India is not contained [1,2]. There is ample evidence that the HIV epidemic has already moved from the high-risk groups via bridge populations into the general population [1]. While HIV prevention efforts have focused largely on high-risk groups in urban areas and along highways (such as sex workers, men-having-sex-with men (MSM), injecting-drug users, and truckers), relatively little attention has been given to rural areas. This is quite surprising, since high-risk behaviour is not restricted to urban areas [3], and 72% of Indians live in rural areas, where the estimated HIV prevalence (0.25%) is only slightly lower than in urban areas (0.35%) [2,4]. Accordingly, as 64% of HIV infections in India are now being reported from rural areas, where awareness is found to be dangerously low, they have become a new battleground of HIV [5-8].

This problem is exemplified in rural districts such as Perambalur, in the south-Indian state of Tamil Nadu. With a rural population of 87%, Perambalur district has the highest percentage of rural population among all districts in Tamil Nadu (with an overall 56% rural population)[4]. Based on population size, Perambalur is the 5th smallest of the 29 districts of Tamil Nadu (At the time of the program, Perambalur district was a fusion of Perambalur and Ariyalur districts and therefore respective population numbers of Census 2001 were combined); however, it ranked 15th in number of cumulative AIDS cases [9]. This high infection rate is possibly due to a combination of factors.

Low awareness and high stigma regarding HIV and sex/sexuality-related issues is fuelled by socio-economic conditions of poverty, low literacy and cultural traditions that consider sexual topics taboo [10]. Basic literacy, at 66% (78% for men, 54% for women) in Perambalur district, is the 3rd lowest in the state [11]. Spousal communication about sex and sexual health is limited. Due to gender inequity, women have little or no ability to negotiate safe sex and are left vulnerable to infection, violence and stigma [12,13]. Although official reports stated that HIV awareness in rural areas of Tamil Nadu had increased in 1997 to 94.4% [14], a 2001–2002 survey performed by a network of nongovernmental organizations (NGOs) revealed the level of HIV awareness to be dangerously low in this district [10]. Of 10 000 respondents (stratified by occupation), only 41% had heard about HIV/AIDS. Only 63% of these 'knowing respondents' (26% of the total population) were aware that HIV was transmitted through 'unsafe sex', while 68–74% of 'knowing respondents' wrongly identified touch and sharing the same house or clothing as transmission routes [10].

Geographically, the national highway that connects the state capital of Chennai to Madurai bisects Perambalur and makes this district a stopover for truckers seeking casual sex [15]. The high spatial dispersion of the population of this district (1.2 million people; 3690 square kilometres) impedes distribution of correct information [4]. Many villages lack public transportation and can only be reached by NGO staff by walking, bicycle or motorbike. In addition, due to the drought-prone nature of this district, there are high seasonal migration patterns with men leaving their families behind in the villages for long periods of time to seek work in cities (where they are more likely to engage in high-risk behaviour). Some women turn to casual sex work as a way to support their children while their spouses are away ('personal communications'). But unlike the red-light districts in cities, much of this sex work is hidden and therefore more difficult to reach with targeted awareness programs.

At the village level, the basic health-care infrastructure is minimal, leading to most villagers seeking initial medical assistance from local unlicensed medical practitioners (including 'quacks')[10]. Travel expenses often constitute an insurmountable barrier for timely access to professional assistance in district headquarter hospitals, VCT centres or other urban healthcare facilities [16].

These conditions, which resemble those of many rural areas in India and other developing countries, posed extra challenges to implement HIV programs. This paper describes the Perambalur Education and Prevention Program (PEPP), that was launched in January 2005 to develop and investigate the feasibility of a HIV peer education model for such rural communities.

## Methods

### Theoretical framework for the program

**Figure 1:**
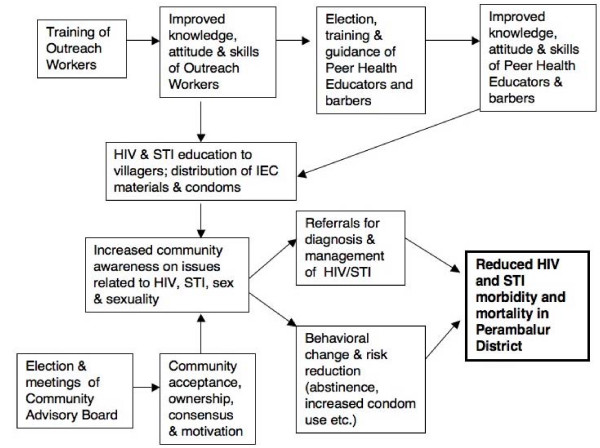
**Impact theory of PEPP**. PEPP was designed to promote community awareness, empowerment and participation through a combination of activities, including participatory trainings for peer educators, outreach educational activities with distribution of IEC materials, and referrals for diagnosis and treatment of HIV and STI. The trained NGO staff (Outreach Workers) guided and assisted the women's SHG leaders (Peer Health Educators) and barbers. The long-term goal (which was beyond the scope of the evaluation plan of this one-year program) was to reduce the morbidity and mortality of HIV and STI in the district.

PEPP was based on Rothman and Tropman's Model of Community Organization [17], where change is sought through participation of a broad cross-section of the community members (including the use of existing social networks [18]), who attempt to identify and solve their own problems. The key concepts of such program include increased empowerment, participation and community competence. Accordingly, PEPP was designed as a pilot project to address these issues through a combination of activities, including participatory trainings for peer educators, outreach educational activities with distribution of IEC materials, and referrals for diagnosis and treatment of HIV and STI (see Figure 1).

### NGO network formation & PEPP

Six established developmental NGOs (READ, INDOTRUST, OSAI, SUBIKSHA, DMI and PAT), active in Perambalur district, had previously formed a network ("AIM network") that had prior experience with small-scale HIV programs [10]. These NGO's, with similar missions to help the underprivileged in their areas, had previously established a good community rapport through a variety of ongoing socio-economic and educational development programs (including women self-help groups, schools and skill-training programs), which were considered to be a good foundation on which to build PEPP. PEPP had a period of one year, from January to December 2005.

### Creation of a Community Advisory Board (CAB)

To promote community acceptance and ownership, the NGO leaders formed a 15-member CAB representing a broad cross-section of the community, including a doctor, a nurse, a social worker, a lawyer, a school principal, a person living with HIV/AIDS (PLHIV), a barber, and leaders of women's SHG, youth groups and disability groups. The CAB had 2 formal meetings during the program period; members attended the programs at the village level to provide input.

### Development of information, education and communication (IEC) materials

**Figure 2:**
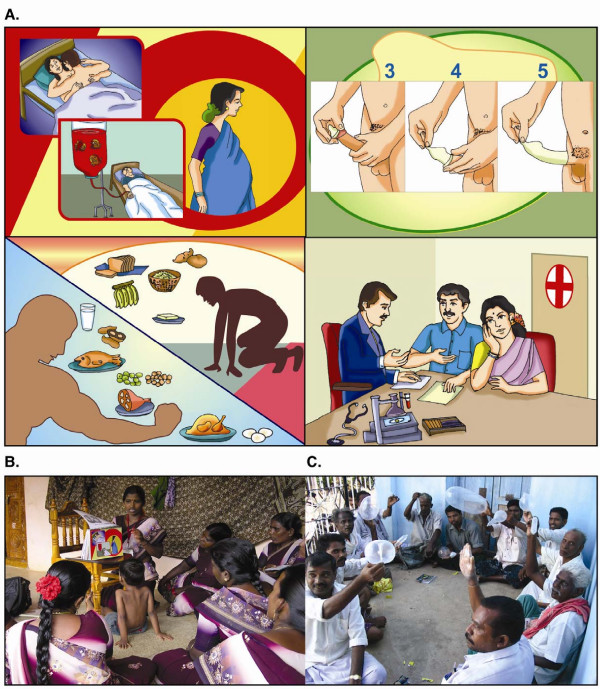
**PEPP activities: IEC development, training and outreach activities to educate low-literacy populations**. A. To educate low-literacy populations and encourage dialogue, cartoons were developed with simple information on HIV transmission, prevention, support and care. The cartoons were used to prepare flipcharts (with *Tamil *and English text on the backside) [20], small booklets and one-page pamphlets to distribute to the public. B. A trained female Peer Health Educator uses the flipcharts to educate a women's self-help group on HIV and AIDS. C. As part of their training, barbers do games to overcome their stigma and fear about condoms.

Due to the low literacy in the community, cartoon-based IEC materials with simple messages on HIV/AIDS were developed. The contents were based on the 'Health Belief Model' [19], to teach people about their own personal susceptibility to HIV/AIDS, the impact of HIV infection on their lives, ways they can reduce their own risk, and strategies to overcome barriers to individual change. The IEC materials addressed sensitive but important topics, such as cartoons to depict the relative risk of different sexual acts. The materials were designed to offer people practical and culturally appropriate choices consistent with the ABC approach to lower their risk of sexual HIV transmission (e.g., masturbation as a form of abstinence; Kama Sutra (i.e., the exploration of different sexual techniques) to avoid boredom in a monogamous relationship, etc.). The cartoons were used to prepare 2 sets of flipcharts titled "Myths and Facts about HIV/AIDS" (see Figure 2 for examples) [20], pocket-size 40-page booklets and one-page fact sheets on HIV/AIDS. The materials were pre-tested in the rural communities among women self-help groups and PLHIV, and by HIV counsellors at the VCT centre of the Government Hospital for Thoracic Medicine (Tambaram, Tamil Nadu). In addition, 11 different designs of stickers with slogans on HIV/AIDS in the local language (*Tamil*) were produced.

### Selection, training and appointment of staff for subsequent outreach activities

Three categories of staff were selected and successfully trained on issues related to HIV/AIDS, sex and sexuality: NGO field staff (Outreach Workers; OW), women's SHG leaders (Peer Health Educators; PHE), and barbers. The reason to include barbers was that most rural men visit barber shops, which are a typically all-male environment where sexual topics are often discussed.

To select the PHE, 9 meetings of women's SHG leaders (for a total of 480 leaders) were first held in March 2005 to introduce and explain the program to them, and 153 candidates were selected. To select the barber trainees, the NGO staff first contacted the Barbers' Association at Andimadam for guidance, which in turn nominated 75 barbers; the staff introduced the PEPP program to them and invited them for subsequent training. The duration of the training was six, four and two days for OW, PHE and barbers, respectively. All trainings were performed in the native language of Tamil. While the OW were paid staff employed by their respective NGOs, the PHE and barbers received a modest stipend for undergoing the training (to offset loss in daily wages). Each training program included pre- and post-test questionnaires to evaluate the change in level of knowledge after the training. Only those who passed their respective post-tests with sufficient scores were appointed as educators; during an inauguration ceremony (September 2, 2005), they received an official certificate and a 'Health Education Kit', namely a bag that contained flipcharts, booklets, pamphlets, stickers, a plastic box for condoms, a waterproof folder, referral slips, reporting forms, a set of writing materials and stationery, a water bottle, and (except for the barbers) an identification badge and business cards.

The appointed educators promoted HIV awareness through a variety of programs. In addition, a Cultural Team (previously formed by the OW of READ [10]) performed street theatre with acts that illustrated the modes of HIV transmission, the impact of the disease on the body's defences of the infected person, and ultimately on his/her family; songs, folk dances and humorous skits were used to engage, entertain and educate the audience.

The PHE received a modest stipend (approximately nine USA dollars per month) for their programs with women's SHG's. The barbers did not receive any direct monetary stipend for their participation in PEPP, but near the end of the grant period, were rewarded for their ongoing efforts with a barber kit (containing barbershop supplies).

Throughout the program period, the 20 OW met once a month with the program supervisor. The 102 PHE also met once monthly (in 4 batches of approximately 25 women). These meetings were held to review the ongoing activities, clarify doubts, resolve any problems, collect the recorded data, and plan upcoming activities. The OW visited barbers regularly in their barbershop to supply more educational materials and condoms, and to answer any questions.

### Referral system

The NGOs had previously compiled a directory of healthcare services available in the district. Referral slips were used to direct people to reliable healthcare providers for voluntary counselling and testing (VCT) of HIV, and diagnosis and treatment of sexually transmitted infections (STI). Referral for VCT was done to four government VCT centres, which were within one hour of travel time of the target villages, and where clients paid ten indian rupees (approximately $0.25) for HIV testing. PLHIV were referred to government hospitals for free medications to treat opportunistic infections and if eligible, antiretroviral medications; they were also encouraged to join the PLHIV network for additional support and care services (including counselling, nutritional support, and access to loans for micro-enterprise development).

### Monitoring and evaluation

The evaluation of PEPP involved the collection and analysis of both quantitative and qualitative data. Triangulation of data was ensured by utilizing multiple data sources, including monitoring statistics. Pre- and post-test questionnaires with multiple-choice questions were collected for (i) all training programs of the 3 categories of peer educators, and (ii) 198 SHG that were educated by the PHE during the outreach activities. All pre- and post-test questionnaire data were entered and analyzed using Microsoft Excel: Mac 2004 software; paired t test *p *values < 0.05 were considered statistically significant. After the one-year program period, five post-intervention focus group interviews were conducted from 2–10 January, 2006; two discussions were held with OW (n = 9 each), two with PHE (n = 8, n = 9), and one with barbers (n = 10). Focus group discussions used questions on several key themes: IEC materials, program evaluation (including training and outreach activities), HIV in the district (changes in awareness, attitudes, community involvement), and recommendations for future programs. Focus group discussions were recorded on a digital voice recorder; an external consultant translated them from Tamil to English. The transcripts were analyzed by reorganization based on common themes. Evaluation staff also conducted five key informant interviews.

## Results

### Selection, training and appointment of educator staff

Three categories of educators were trained in their native *Tamil *language. A common theme was that prior to the training, many misconceptions persisted. Many trainees were initially very shy and hesitant to discuss sex-related issues, so participatory activities and fun games were used to help them gradually overcome their fears and build confidence (see Figure 2).

#### (i) Training of NGO field staff to become Outreach Workers

In February 2005, external resource trainers provided a six-day training to 30 NGO field staff. Pre- and post-test questionnaires revealed average scores of 46% and 82%, respectively (p < 0.0001; 2-tailed paired t-test). Twenty five field staff passed the training with post-test scores of ≥ 85%, and 20 were appointed as PEPP Outreach Workers (OW). A supplemental three days of training on counselling was given in June 2005.

#### (ii) Training of women's self-help group leaders to become Peer Health Educators

Following the selection of 153 SHG leaders, the training was conducted in 6 batches, each consisting of a four-day training program. The average pre-test score was 43%, and only one woman scored more than 70%. Sixteen women dropped out during the training program because of objections to its sexual content. Of the remaining 137 who completed the training, the average pre- and post-test scores were 42% and 82%, respectively (p < 0.0001; 2-tailed paired t-test). Of these 137 women, 119 women passed the training with a post-test score of ≥ 70% score (mean score 86%), and 102 of them were employed as official Peer Health Educators (PHE).

#### (iii) Selection and Training of Barbers

Because most of the 75 barber trainees were illiterate, pre- and post-training tests were administered orally by READ staff in individual format. Awareness prior to training was low (mean score 25%); for example, 82% of barbers were not aware that unprotected anal sex posed a risk of HIV transmission. Because an initial one-day group training (held in April 2005) did not raise their scores sufficiently (mean score 47%), supplemental training was conducted in smaller groups, and a second one-day group training was performed in July 2005. Following this second training, 52 of the 79 attendees had sufficient post-test scores (≥ 70%) to qualify as peer educators for PEPP.

### PEPP field activities to promote HIV/AIDS awareness for self-help groups, other community groups and the general public

As described below, the different categories of educators conducted a variety of outreach programs to disseminate information on HIV/AIDS-related issues. Approximately 23 000 HIV booklets, 27 000 one-page HIV fact sheets, 12 000 stickers and 69 000 condoms were distributed during 2051 interactive HIV awareness programs and one-on-one communications. Although some overlap in attendance between the different programs occurred, it is estimated that at least 30 000 persons were directly exposed to HIV information through these outreach programs.

#### (i) Female Peer Health Educators

The 102 female PHE, with assistance of the OW, conducted HIV awareness programs for 607 women's SHG (at least 2 programs per PHE per month), with a total coverage of approximately 9000 women. PHE typically visited each women's SHG three times during the term of the program; the first session focused on sexual anatomy, reproduction and STI, while the subsequent two sessions utilized the IEC materials to discuss HIV/AIDS (Figure 2). To evaluate the HIV education program, group pre- and post-test questionnaires were administered by the PHE to 198 women's SHG before the second and after the third session, respectively. The average pre- and post-test scores were 57 and 75%, respectively (two-tailed paired t test, p < 0.0001).

#### (ii) Barbers as male peer educators

A novel approach to HIV peer education in this area involved utilizing barbers as peer educators. Barbershops are typically an all-male space and discussions often centre around sex. The 52 trained PEPP barbers displayed their training certificate, the HIV flip-charts and HIV pamphlets in their barbershop (which was a 1- or 2-chair roadside shop or stall). Barbers demonstrated condom use on wooden models, provided free condoms and booklets to their clients, and answered questions on HIV/AIDS. Initially each barber was provided with a free blade-holder and a set of disposable blades; after that, they voluntarily purchased disposable blades and reported using a new blade for each customer.

#### (iii) Outreach Workers

The 20 OW, in addition to supervising and guiding the female PHE and barbers, also conducted 47 presentations to the general public and 218 programs for local community groups (e.g., youth groups, farmers groups, and factory workers), which reached an estimated 17 500 people. The OW also performed 51 street theatre programs, with an estimated total attendance of approximately 15 000 people. They also organized 37 HIV awareness rallies with the local communities (scheduled around 1 December 2005, World AIDS Day).

### Referrals and support & care services

While some referrals were given verbally, written records document 2844 referrals. At least 45% of the referrals that were done via referral slips resulted in visits, based on collection of ticket stubs from the participating healthcare centres. An estimated 75% of the referrals were for HIV voluntary counselling and testing (VCT); the remainder was for STI and other medical problems; 118 persons were newly diagnosed as PLHIV. The OW also provided counselling to individuals and families affected by HIV. A total of 129 people, including persons identified as PLHIV prior to PEPP, were referred to the Government Hospital for Thoracic Medicine at Tambaram (near Chennai), which at that time was the main government hospital in the state of Tamil Nadu that offered some free medical care for PLHIV. Travel costs were covered by PEPP. The PLHIV network that was started in 2002 by READ grew in 2005 from seventeen to more than 100 members because of the increased uptake of VCT. As of January 2006, 88 members of this network travelled regularly to the Government Hospital for Thoracic Medicine in Tambaram, and twelve members (including five children) were receiving antiretroviral drugs. Thirty members of the network had received a loan ($50 to $100) from a revolving loan fund to start an income-generating activity.

### Qualitative evaluation using focus group discussions and key informant interviews

In January 2006, focus group discussions were held among the different groups of educators; in addition, key informant interviews were conducted with members of the general public (self-help group members and barbershop customers). These discussions revealed that as the program progressed, the trained peer educators and the general public gradually gained confidence in talking more openly about sensitive topics and expressed satisfaction in noticing changes in attitudes and risk behaviors.

"*Even the mere utterance of the word HIV/AIDS was a taboo before. And now we are clear about that, and we are able to clear the doubts of others also on HIV/AIDS* (PHE)."

"*In the beginning, our customers felt very awkward to see the penis model placed at our shop. That attitude is changed now and they try condom demonstrations by themselves using the penis models*;" "*Many learned the correct method of using condoms; many have stopped involving in multi-partner sex* (Barbers)."

"*Now after our awareness education ...many abstained from getting injections for their common diseases. In case they cannot avoid injection, they buy disposable syringes and insist that the doctors use them* (PHE)."

"*Before I attended the program, I treated HIV-infected people badly. Now I understand, I talk with them, I go out with them* (SHG member)."

A theme that emerged in all focus group discussions and key informant interviews was the need for HIV education for students and youth.

"*Girls and boys must learn. When we were young, we received no education, we had no access* (SHG member)."

"*More viewers are from the student community than elders*." "*Every one who used those materials stated that they had learned a lot from the materials* (Barbers)."

"*Teachers were not the best choice to educate students on sex/sexuality and HIV/AIDS because students, out of respect or fear for teachers will not come forward to seek clarification from them*;" "*School students asked us to provide training to them so that they can pass the information to their fellow students* (OW)."

The educators acknowledged that the educational cartoons contributed to the success of the program. Although the community response to the materials was favourable, some of the graphics related to prevention of sexual transmission and different sexual acts were, not unexpectedly, a topic of discussion, and evoked varied responses ranging from disbelief to further interest. The far majority of women and men did not criticize the cartoons but recognized its function in disseminating health information and encouraging sexual dialogue:

"*The materials we published were the best because they reach everyone, both literates and illiterates* (OW)."

"*They were taken aback because they have not seen such pictures before*;" "*They could not understand the different types of sexual play – vaginal, oral, anal and non-penetrative sex- in fact, they wonder about those different types. They used to ask whether the different illustrated sexual acts are possible*;" "*They commented, the animals which are considered lower to human being, have only one type of sexual play but the human beings have so many different types, why*?" "*In this situation we developed dialogue with them and slowly removed the sensitivity. Fortunately some matured audience came to our rescue and convinced the rest of the audience that these are part of our daily lives and we don't need to be too sensitive* (PHE)."

Focus group discussions also revealed that the program benefited the female PHE in other ways. "*I was looked down when I went for PEPP training in the beginning. But after my interaction with them on the subject I learned in the training, their outlook changed. And now they are very eager to learn new information from me*;" "*We surprised people who ask us how an ignorant woman is able to speak on different subjects so clearly*;" "*The people are fascinated by our new status with a kit bag, ID card and different social identity... Many ask us to get them also a similar job* (PHE)."

Initially, NGO staff acknowledged the possibility that barbers who participated in PEPP may be stigmatized or lose customers, but focus group discussions revealed that this was not the case. "*This work does not affect our profession and we are happy and proud to do this service... (We are) able to answer even intricate and difficult questions on HIV/AIDS; questions of educated and school learning people also*;" "*It is generally stated that whenever one wants to know about male (sexual health) one has to refer to the barbers*;" "*Discussions surrounding sex were very free and frank; ordinary people will not speak and discuss freely with doctors* (Barbers)."

As the general public gained more awareness on blood-borne transmission of HIV and other diseases, the PEPP barbers, who began using disposable razor blades after their training, reported an increase in customers.

## Discussion

The current report highlights the HIV-related issues that affect rural communities in Perambalur District, Southern-India, and illustrates the development and field-testing of a model that addresses these problems by incorporating HIV awareness programs in established networks and empowering local men and women with peer education skills and educational materials. The lessons learnt from this program apply to many other rural areas that are in need of similar activities.

Although some reports continue to claim that HIV awareness in India and particularly in Tamil Nadu is high [14], our pre-training sample of rural barbers and SHG members revealed many recurrent misconceptions, even for basic questions on how HIV is and is not transmitted. This was especially true for barbers, who were not a specific target audience of our previous small-scale HIV awareness programs [10], but whose level of education and literacy is likely representative of a large section of the male population in rural areas. The poor HIV awareness among low-literacy populations in rural areas is less surprising in light of the results of a recent survey that revealed similarly low HIV awareness among Indian lawmakers [21]. Such findings suggest that the current HIV awareness programs, which focus mostly on high-risk groups, are not able to convey accurate or comprehensive awareness to the rest of the population, leaving them vulnerable to HIV infection and likely to harbor unnecessary fears and stigma against PLHIV. Mass media campaigns (such as radio, television, and posters) focus usually on a limited spectrum of messages about sex and condom use. More comprehensive sources of HIV information (such as brochures) are often available at the larger district hospitals but usually do not reach the healthcare facilities at the village level. Additionally, in the absence of a trained educator or counsellor who has time to provide a complete explanation, many people are shy or afraid to ask HIV- or sex-related questions, and such information does not reach people with low literacy [5,22,23]. Rural women are especially vulnerable to infection, as many of them are trapped in socio-cultural conditions of subordination, are confined largely to their village and immediate surroundings, and are denied access to information, medical treatment, or the ability to protect themselves against potentially unsafe sex with their husband.

HIV peer education programs are an appropriate way to break the silence and have been successful in many countries, because peer education can provide culturally appropriate and acceptable information, and its community-based nature promotes sustainability at relatively low cost. Peer education programs in India have focused mostly on the high-risk groups and urban areas, such as sex workers (e.g., Sonagachi in Kolkata [24]), MSM populations, and university students [25,26], but very few examples have been documented in rural areas. A program in rural Karnataka found that peer education programs can be effective to launch mass awareness campaigns, but that sustainability after the project period (and in the absence of external funding) was very limited unless peer educators were affiliated with village level institutions that had a larger portfolio of leadership building and community services [27].

PEPP was designed to test the feasibility of a peer education model aimed at educating and empowering low-literacy rural communities in Perambalur district. The main outputs of PEPP were (i) improved community awareness on HIV/AIDS, (ii) referrals, and (iii) distribution of educational materials and condoms. As PEPP was a one-year pilot project with limited budget, quantitative measurements of changes in sexual behaviour and changes in HIV incidence rates were beyond the scope of this project. However, a recent study in Africa, aimed at evaluating the efficacy of a novel HIV intervention strategy (pre-exposure drug prophylaxis) in high-risk groups found an unexpectedly low infection rate even in the placebo group due to improved education, counselling, and provision of condoms, relative to what was available prior to the trial [28]; these components were also the corner stones of PEPP.

PEPP demonstrated that forming a peer education network that is integrated with local developmental programs and established community-based organizations is an effective way to disseminate culturally appropriate and comprehensive information about HIV/AIDS and promote health-seeking behaviour among low-literacy communities in this rural Indian district. In our program, the women's SHG, formed with the primary goal of socio-economic empowerment through micro-finance activities, provided a good forum to select motivated leaders to be trained as PHE who subsequently educated members of their own and of adjacent SHG. These women, who were already acquiring leadership qualities and social recognition in their communities, developed the skills and confidence to gradually talk openly about sensitive and sexually explicit topics, something that may otherwise have been an insurmountable barrier. Their newly found role as promoters of public health became a source of pride and additional social recognition, which may further contribute to the sustainability of the peer dialogue and communication on HIV- and AIDS-related issues. A recent study from South Africa indicated that a combined micro-finance and gender/HIV training curriculum of women reduced intimate-partner violence [29]; although it remains to be determined whether similar effects occurred in the women's SHG that participated in PEPP, a decrease in intimate-partner violence may further contribute to a reduced risk environment of these women for HIV infection.

To reach the male population, our program trained barbers as HIV peer educators. Giving barbers a role in public health is not new. Prior to the development of a separate medical profession, barbers fulfilled the traditional role of healers and surgeons [30-32]. Several other organizations in India have previously used barbers as HIV peer educators [33,34]. Our program confirmed that with proper training and equipped with good materials, barbers in rural Perambalur district can be successful peer educators. The PEPP barbers did not report stigma from customers in their new role as promoters of better sexual health. Instead, some barbers commented that they attracted more customers, possibly also because of the introduction of disposable razor blades. This is particularly significant as barbers had no (other) financial incentive to participate in the program.

Another theme that emerged in all focus group discussions was the request for HIV education for students and youth. Although the National AIDS Control Organization (NACO) lists 'School AIDS Education Programmes' [35] as one of four key areas recommended for partnering with NGOs and programs have been implemented in Tamil Nadu to educate high school headmasters on HIV/AIDS, PEPP findings suggest that HIV education in the school system in 2005 did not clarify all the students' doubts. This was likely because students were too shy to openly ask sensitive questions. This indicates that more attention needs to be given to train peer educators among students instead of the traditional lecture-in-a-classroom model of HIV education.

The careful development of cartoon-based IEC materials was an important component of PEPP, because low-literacy communities can only learn how to cope with HIV if provided with clear and easily understandable information. Our prior search for available educational materials had revealed a lot of materials that were vague, incomplete, too medical, or that required technologies (e.g., video-players and televisions) that are unavailable in many rural areas where access to electricity is limited. Although the educational materials of PEPP had some explicit cartoons and messages, the consensus among all groups of educators was that the quality, depth and comprehensiveness of the educational materials contributed significantly to the success of the program. Since the initiation of PEPP, organizations that are active in other states of India or other countries have expressed eager interest in translating or adapting the PEPP IEC materials; this suggests that 25 years into the HIV epidemic, access to simple and practical educational materials on HIV/AIDS is still deficient in many regions of the world. Accordingly, more attention should be given by funding agencies to support local organizations with the design and/or distribution of materials that convey simple, comprehensive messages on HIV and AIDS that fit the needs of their target communities.

The program promoted better HIV-specific health awareness and health-seeking behaviour of the villagers. However, the ethical dilemmas associated with promoting VCT in remote areas with limited access to treatment, and where rampant poverty limits transportation to urban healthcare centres, became apparent. Although PEPP covered travel expenses of many villagers to nearby VCT centres and of PLHIV to the Government Hospital for Thoracic Medicine in Tambaram to get free government-sponsored HIV medications, coverage of such travel expenses of PLHIV (approximately USD 7 for a round-trip, equivalent to a week's salary) became problematic after the expiration of the 1-year grant period. This was especially because many new PLHIV had joined the network during the short period. Some PLHIV's poor health status did not permit them to undertake the long journey (6-hour one-way trip by bus), and they passed away at home [16]. In addition, PLHIV reported stigma from some local hospital employees. Thus, structural interventions, including better medical infrastructure, and more training of all hospital staff on HIV-related issues are needed to ensure that PLHIV in rural areas have access to unstigmatized medical care and support services closer to home.

## Conclusion

Using established networks (such as community-based organizations) and training women's SHG leaders and barbers as peer educators is an effective and culturally appropriate way to improve communication, disseminate comprehensive information on HIV/AIDS and provide referrals in low-literacy communities. In many remote rural communities, there are ordinary people with little or no academic credentials, but who with proper training and equipped with appropriate materials can be empowered to cross their personal boundaries and become extraordinary peer educators and voices for change in their own communities. The current study indicates that more effort is warranted to tap into this large unrecognized force. National and international agencies should dedicate more funding to expand and replicate similar peer education models in many other rural areas that are in urgent need of similar activities to avert an increase in HIV prevalence.

## Competing interests

The authors declare that they have no competing interests.

## Authors' contributions

KVR, PM, KK, VC and DS participated in the initial concept, the design of the study and the development of the IEC materials. KVR assisted in data analysis and drafted the manuscript. MR designed the monitoring and evaluation plan and analyzed the data. VC provided training to Outreach Workers. DS coordinated all activities and data collection. All authors read and approved the final manuscript.
